# *Trichinella*-Derived Extracellular Vesicles: Implications and Future Prospects

**DOI:** 10.3390/pathogens15020136

**Published:** 2026-01-26

**Authors:** Dalia S. Ashour, Ahmad A. Othman, Hager S. Zoghroban

**Affiliations:** Medical Parasitology Department, Faculty of Medicine, Tanta University, Tanta 31527, Egypt

**Keywords:** *Trichinella spiralis*, extracellular vesicles, miRNA, immunomodulation, biomarkers, vaccine candidate

## Abstract

Parasite extracellular vesicles (EVs) are concise and versatile messages for parasite–parasite and parasite–host crosstalk. These vesicles are loaded with a cargo of diverse heterogeneous molecules, some of which are of potent immunomodulatory nature, and others have specific functions. Those EVs carrying the *Trichinella* signature are no exception. They play pivotal roles in the establishment of the parasite inside its niche within the host, ensuring better survival for both. They can also serve as biomarkers for diagnosis, follow-up, and prognosis of *Trichinella* infection. Owing to their immunogenicity and durability, they are excellent candidates for vaccine development. Moreover, enriched by the parasite’s elements, these intriguing EVs could protect the host from a wide array of inflammatory conditions associated with immune dysregulation such as inflammatory bowel disease and airway hyperreactivity, as evidenced by well-conducted experimental preclinical research. In sum, the potentials of *Trichinella* EVs seem enormous, awaiting only to be better characterized and conditioned for use in diagnostics and therapeutics. Detailed proteomic and transcriptomic analyses of the nature of these parasite-derived elements could provide invaluable insights into parasite biology and its interplay with the host at the same time.

## 1. Introduction

Extracellular vesicles (EVs) are nano-sized structures surrounded by a lipid bilayer, released by all cell types during physiological and pathological conditions. As crucial mediators of intracellular communication, EVs transfer a complex set of biomolecules including proteins, nucleic acids, and lipids between cells [1,2].

Parasites have evolved complex life cycles where intercellular communication is essential. Over the last few years, the study of EVs has revolutionized our understanding of how pathogens interact with the host in favor of their persistence. Moreover, advances in the comprehension of the biological roles of EVs have increased our knowledge about the development of several pathologies and opened up new avenues for the development of new diagnostic methods and new vaccines for parasitic infections [3].

Most parasites at some stages in their life cycle rely on the ability to communicate in parasite–parasite as well as in parasite–host interactions. Previous studies in this area have focused mainly on the soluble proteins secreted by parasites and their role in pathogenesis and modulation of the host immune response (reviewed in McKerrow et al. [4] and McSorley et al. [5]). Parasite-derived EVs are particularly interesting components of excretory–secretory (ES) products. They contain parasite-specific information for uptake and integration into other cells without direct contact between cells [6].

## 2. Biogenesis and Kinetics of EVs

EVs are membranous compartments formed through four stages: (1) initiation (selection of molecules such as nucleic acids, proteins, and others); (2) endocytosis (endocytosis of extracellular material by the plasma membrane invagination and intraluminal vesicle (ILV) formation); (3) multivesicular-like body (MVB) formation (formed through aggregation of various ILVs in a large body); and (4) secretion in the extracellular environment (upon exocytosis of MVBs through fusion with the plasma membrane). Vesicles generated in multivesicular endosomes are called exosomes. Other vesicles can be formed at the plasma membrane by direct budding into the extracellular space, giving rise to microvesicles and membrane particles [7,8]. However, in response to the uncertainty in many instances of their biogenesis, Minimal Information for Studies of Extracellular Vesicles (MISEV) guidelines have classified EVs into ‘large EVs’ (>200 nm) or ‘small EVs’ (˂200 nm) [9]. Furthermore, EVs have been classified based on their size into different types: exosomes, 30–120 nm; microvesicles, 150–1000 nm; apoptotic bodies/vesicles, 100–5000 nm; migrasomes, 500–3000 nm; large oncosomes, 1–10 μm; and exophers, 3.5–4 μm [10,11].

Much interest has been paid to EVs contents. EVs contain a varied series of bioactive cargos consisting of proteins, lipids, and diverse genetic materials, including DNAs, RNAs, mRNAs, miRNAs, and circRNAs [12]. Protein is the major component investigated in almost all studies of EVs. It includes two main categories: the specific components that exhibit unique protein signatures (cell-specific and varying depending on the cell of origin) [13] and the universal components, such as membrane transport and fusion-related proteins (like Rab, GTPases) [14], heat shock proteins (like HSP70, HSP90), annexins, and the lysobisphosphatidic acid (LBPA)-binding protein Alix [15]. Several adhesion molecules, such as CD146, CD9, CD18, and CD11 [16], and tetraspanin, including CD9, CD63, CD81, and CD82 [10], are also carried by EVs.

It is noteworthy that in addition to parasite proteins, EVs also contain host-derived proteins indicating the cross communication between the parasite and the host. For example, Marcilla et al. [17] identified 36 different host proteins in the EVs from *Echinostoma caproni* adults. In *Fasciola hepatica* EVs, they identified 19 host proteins. Similarly, parasite- and host-derived proteins were reported in *Echinococcus multilocularis* [18] and in intracellular parasites such as *Plasmodium falciparum* [19]. The most abundant host-derived proteins in parasite EVs were immunoglobulins, complement, and metabolic enzymes. It has been suggested that these host proteins may be packed into the parasite EVs during release into the extracellular space [18].

Moreover, EVs may carry bioactive lipids to recipient cells [20,21]. They contain 2–3 times the amount of cholesterol and glycosphingolipids relative to that in their progenitor cells, which vary among different cell origins [22]. However, only a few studies have yet explored the lipid content of EVs in parasites, and their specific role remains to be elucidated [23,24]. The current knowledge of helminth-derived EV molecular contents is found in a review by Sotillo et al. [25].

Once released from the secreting cells, EVs recognize and attach to their target cells through three possible ways: (1) endocytosis: the endocytosed EVs could fuse with the limiting membrane of the endosome; (2) receptor-ligand interaction: the binding of secreted vesicles to the surface of a recipient cell involves interactions between EV surface proteins (such as integrins, tetraspanins, proteoglycans, lectins, and immunoglobulins) and cellular receptors (such as intercellular adhesion molecule 1 (ICAM1), phosphatidylserine, and other unknown receptors); and (3) direct fusion of EVs with the plasma membrane. Finally, incorporation of proteins from the EV membrane into the plasma membrane and the release of EV contents into the cytoplasm of the recipient cell occur. The transfer of their content occurs as rapidly as 15 min after EV fusion with the target cells [26,27].

Here, we focus on the nematode *Trichinella spiralis*, as the research interest in *T. spiralis* EVs (*Ts*-EVs) has surged in the last few years. *T. spiralis* is a zoonotic parasite with a worldwide distribution acquired by the consumption of raw or undercooked meat infected with *T. spiralis* infective larvae (L1). It infects a wide variety of hosts, notably domestic pigs, rodents, and humans [28]. *T. spiralis* is distinctive among helminths, as it completes its entire life cycle within a single host. Following infection, the larvae are released in the stomach, invade the small intestinal mucosa, mature into adult worms (AWs), and reproduce. The resulting newborn larvae (NBL) disseminate through the lymphatic and circulatory systems to the striated muscles. Upon invasion of muscle cells, *T. spiralis* transforms them into nurse cells surrounded by a collagen capsule [29]. *T. spiralis* is a very successful parasite capable of producing long-term infections. The virulence of *T. spiralis* is attributed to its ability to manipulate the host immune responses aided by the release of a diverse array of functional proteins, such as heat shock proteins, proteinases, protein kinases, glycosidases, and EVs that promote invasion and long-term persistence [29,30,31].

**Figure 1:**
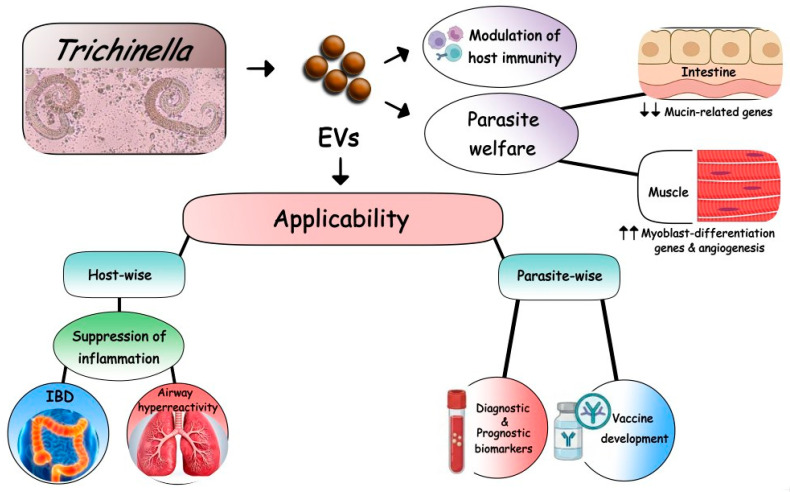
Schematic representation of the multifaceted role of *Trichinella* EVs in host–parasite interactions and their potential clinical applications. *Trichinella spiralis* releases EVs that actively modulate the host immunity, creating a tolerogenic environment that is beneficial for the host and the parasite. *Ts*-EVs act as key mediators for parasite survival. In the intestine, *Ts*-EVs suppress mucin-related genes to compromise the mucosal barrier. In the muscles, they upregulate myoblast-differentiation genes and promote angiogenesis, essential processes for the formation of the protective “nurse cells”. EVs, extracellular vesicles; IBD, inflammatory bowel diseases.

Despite much interest, information on the role of *Ts*-EVs in parasite–host communication is still limited. Therefore, in this review, we aim to demonstrate our current knowledge of *Ts*-EVs as well as their role in host–parasite interactions. Furthermore, implications of *Ts*-EVs as diagnostic biomarkers, novel potential therapeutic targets, and protective agents are discussed (outlined in Figure 1).

## 3. Isolation and Characterization of *Trichinella spiralis* EVs (*Ts*-EVs)

Kosanović et al. [30] submitted the first report on the production of EVs by *T. spiralis* muscle larvae (*Ts*-ML-EVs). *Ts*-EVs have been successfully isolated and characterized from the conditioned medium of *T. spiralis* muscle larvae (ML) [30,32,33,34,35] (summarized in Table 1). Generally, *Ts*-EVs are electron-lucent, intact, and rounded microvesicles with a size ˂ 200 nm. A few studies have reported larger sizes reaching 300 nm [36,37]. Thus, they are described as exosomes or EVs.

Moreover, Taylor et al. [38] quantified the number of EVs secreted by each life stage in vitro. Adult *T. spiralis* secreted about 3.15 × 10^5^ vesicles per parasite per 24 h, in comparison to 1.26 × 10^5^ vesicles per parasite per 24 h for ML. So, relative to their body mass, similar rates of EV production were detected. However, Glamoclija et al. [35] could isolate only 1100 vesicles per ML mostly due to differences in cultivation conditions of the larvae or EV isolation procedures.

Interestingly, Khueangchiangkhwang et al. [39] observed that both *T. spiralis* adult EVs (*Ts*-AW-EVs) and *Ts*-ML-EVs exhibited common functions such as inhibition of macrophage activation and suppression of the classical inflammatory pathways. However, different functions aligning with their specific locations in the host were reported. For example, *Ts*-AW-EVs—but not *Ts*-ML-EVs—significantly downregulated mRNA expression of mucin-related genes, e.g., *MUC1*, *MUC5AC*, trefoil factor 3 (*Tff3*), and chloride channel accessory 3 (*Clca3*). On the other hand, *Ts*-ML-EVs were uniquely effective at upregulating genes related to myoblast differentiation; including myogenin (*MyoG*) and myosin heavy chain embryonic (*Myh3*) mRNA, compared to *Ts*-AW-EVs-treated cells. These functional divergences might be dependent on their unique molecular cargos. Protein profiling revealed that while the two stages share 168 proteins, *Ts*-AW-EVs and *Ts*-ML-EVs possess 92 and 62 other stage-specific proteins, respectively.

Proteomic analysis of *Ts*-ML-EVs identified 753 proteins distributed throughout several compartments in *T. spiralis* ML, most notably in the plasma membrane, cytoplasm, nucleus, mitochondria, and extracellular regions. The most abundant *Ts*-ML-EV proteins included regulatory proteins of translation and signaling, as well as structural and metabolic enzymes [33]. Furthermore, mass spectrometry (MS)-based proteomics on *T. spiralis* newborn larva EVs (*Ts*-NBL-EVs) identified 231 proteins with similar subcellular distributions [40]. These findings align with the previous proteomic analysis of soluble antigens from *Trichinella* NBL by Hao et al. [41].

Interestingly, Western blotting and MS-based proteomics of NBL-EVs did not detect positive bands for the common proteins detected by *Ts*-ML-EVs (CD63 and CD81). Instead, they detected 14-3-3 protein and enolase (ENO1) [40]. The protein cargo into EVs is subjected to selective packaging before release; therefore, changes in EV protein composition between different developmental stages of *Trichinella* mostly reflect different conditions during release of EVs due to their different niches in the host [42]. Representatives of the most frequently identified protein cargo in *Ts*-EVs and their potential functions are listed in Table 2.

## 4. *Trichinella* EVs Are Key Strategic Tools for Infection

It is worth noting that parasite-derived EVs are involved in the pathogenetic mechanisms of parasitic diseases. Moreover, they have the ability to change the composition and function of the released vesicles during host–parasite interactions, thus mediating the disease [49].

During the early stage of infection, Wang et al. [34] investigated the effect of exosomes derived from *T. spiralis* ML (*Ts*-ML-Exos) on the intestinal epithelial cells in vitro. They observed that *Ts*-ML-Exos were significantly endocytosed in porcine small intestinal epithelial cells (IPEC-J2) within 6 h with a significant time-dependent increase in the number of endocytosed *Ts*-ML-Exos. They showed that *Ts*-ML-Exos decreased IPEC proliferation and could lead to increased levels of cytotoxicity, oxidative stress, and abnormal apoptosis. Moreover, *Ts*-ML-Exos significantly reduced the expression of different tight junction-related genes of *ZO-1*, *CLDN-3*, and *OCLN* at 24 h compared with the controls, leading to interruption of tight junctions and significantly increasing the permeability of the cells. Therefore, *Ts*-ML-Exos damaged gut epithelial integrity and played definite roles in the process of *T. spiralis* invasion inside the host. Previously, these findings were attributed to serine proteases in the *T. spiralis* ES proteins [50]; these proteases were recently detected in *Ts*-ML-EVs via liquid chromatography MS (LC-MS)/MS proteomic analysis [33].

Liu et al. [40] reported that the differentially expressed proteins and miRNAs in *Ts*-NBL-EVs were mainly involved in metabolic, autophagic, and other pathways, suggesting that these EVs may play an important role in evading host immune defense during early *Trichinella* infection. Among them, 14-3-3 protein was detected in *Ts*-NBL-EVs and let-7-5p had the highest expression. *T. spiralis* 14-3-3 protein is an immunodominant antigen during the early stage of *Trichinella* infection. It participates in regulating the growth and development of parasites and the process of invading the host [51], while let-7-5p activates the polarization of host macrophages to M2b type to evade the host’s immune response [37].

In the muscle phase of *T. spiralis* infection, *Ts*-ML-EVs play specific roles in nurse cell formation via the enhancement of myoblast differentiation. *Ts*-ML-EVs injected directly into striated muscle of mice caused a significantly higher expression of *MyoG* and Myogenic Regulatory Factor 4 (*Mrf4*) mRNAs at 2 days after the injection. Moreover, proteomic analysis revealed that *Ts*-ML-EVs contained proteins involved in the angiogenesis and vascular endothelial growth factor (VEGF) signaling pathways, involved in the encapsulation of larvae in the muscles. Thus, *T. spiralis* nurse cell formation and encapsulation in the muscle might be regulated at least in part by these EVs [39]. Therefore, an analysis of the proteins in *Ts*-ML-EVs against those in unencapsulated species (*T. pseudospiralis*) is essential to elucidate the specific cargo and molecular mechanism underlying the process of nurse cell formation.

## 5. MicroRNAs Abound in *Ts*-EVs

MicroRNAs (miRNAs), small non-coding RNAs, are key regulators of gene expression conserved across eukaryotes and involved in the regulation of numerous biological processes, such as cell development, differentiation, proliferation, and apoptosis, and in various diseases [52,53]. Many studies showed that miRNAs regulate the immune system functions and shape both innate and adaptive immunity, including Th1 and Th2 polarization [54,55].

Several species of helminths secrete abundant quantities of miRNAs that play pivotal roles in the host–parasite interaction [56]. Substantial amounts of secreted miRNAs are enclosed within EVs. These miRNAs could be taken up by host cells and potentially contribute to gene regulation with further manipulation of their host’s immune responses, through targeting host mRNAs for degradation or translational repression [56,57], but the exact mechanisms remain unclear.

Encapsulation of miRNAs in EVs protects them from degradation by RNases in the extracellular environment and allows their transport and delivery to host cells. However, some extracellular miRNAs are not enclosed in EVs mostly due to differences in the release mechanisms that can vary depending on the helminth species and life stage. However, the EV-enclosed miRNAs are considered the key for mediating interactions with the host [58,59]. Interestingly, Taylor et al. [38] observed that exonuclease treatment eliminated most of the small RNAs secreted by muscle larvae, yet it had little effect on the small RNAs released by adult worms under the same conditions. Thus, they proposed that adult *T. spiralis* secretes predominantly vesicular miRNAs, while most of the miRNAs secreted by *T. spiralis* ML are not contained within vesicles, suggesting they might be released directly into target cells. Moreover, several candidate proteins were identified in *T. spiralis* AW and ML secretomes that might bind and stabilize miRNAs. Two of them, namely KSRP and TsPUF, were characterized by Brown et al. [60].

Both *Ts*-AW-EVs and *Ts*-ML-EVs contain many miRNAs, such as tsp-miR-1, tsp-miR-100-5p, tsp-miR-125b-5p, tsp-miR-57-5p, and tsp-miR-72-5p, which are highly conserved across various helminth species. Sequencing *Ts*-EVs-miRNAs showed that 54 miRNAs were common between AW and ML. On the other hand, 78 and 43 were AW-specific- and ML-specific-miRNAs, respectively. These unique signatures suggest specialized biological roles. For instance, tsp-miR-36-5p is secreted solely by ML. This specific miRNA likely plays a targeted role in modulating host gene expression during the muscle phase of the infection, a process that remains to be fully characterized [39].

Regarding the potential role of miRNAs in *Ts*-EVs, an array of miRNAs was involved in immunoregulation, angiogenesis, apoptosis, cell communication, and other biological processes, including inflammation [36]. miRNA sequencing detected 64 miRNAs (10 of which are known miRNAs and 54 are unknown miRNAs). Among the known miRNAs of *Ts*-EVs, miRNA-1-3p and let-7-5p were the most highly expressed. *Ts*-miR-1-3p and *Ts*-let-7-5p induce the polarization of M2 macrophages and inhibit the activation of host fibroblasts in muscle cells; thereby, the parasite effectively evades the immune response and maintains a long-term existence necessary for its life cycle progression [37].

To investigate its role during the intestinal phase of *T. spiralis* infection, Wang et al. [61] constructed a miRNA library of *Ts*-Exos and then selected miR-153 for further studies. They demonstrated that miR-153 within *Ts*-Exos might be a key factor in the interaction between *T. spiralis* and host cells that participates in the regulation of various biological cellular processes. miR-153 acts as a proapoptotic factor in porcine intestinal epithelial cells (IPEC-J2) in vitro by inhibiting the target gene *Bcl2* (anti-apoptotic pathway) and increasing the expression of proapoptotic proteins such as Bax, Bad, caspase 9, and caspase 3. Thus, miR-153 interacts with the intestinal epithelial cells and facilitates the invasion of *T. spiralis* larvae through induction of apoptosis and intestinal cellular damage as well as increased oxidative stress levels.

During the muscle phase, *T. spiralis* larvae induce a number of changes in the host muscle cells, involving direct manipulation of gene expression by factors secreted by *T. spiralis* larvae [62], which have been recently shown to include abundant small non-coding RNAs [38]. Among miRNAs enriched in the secreted material by *T. spiralis* ML, a homologue of the mammalian miR-31 was identified [38]. miR-31 has a well-documented role in muscle development by regulating translation of the myogenic determination gene *Myf5*. miR-31 is transcribed at high levels in quiescent satellite cells and is sequestered together with *Myf5* RNA in messenger ribonucleoprotein (mRNP) granules. Following activation of satellite cells during development or after injury, mRNP granules are dissociated, miR-31 is reduced, and *Myf5* mRNA is released from repression, allowing myogenesis to proceed [63]. Thus, miR-31 *T. spiralis* homologue is also predicted to target the *Myf5* gene with further activation and proliferation of satellite cells, then remodel into a nurse cell.

## 6. Applications of *Trichinella*-Derived EVs

Research on EVs of parasites is promising, with many reports about parasite EV proteins at some stage in their life cycle. They are being investigated as new agents for diagnosis and vaccination candidates. Moreover, they may also be useful tools to ameliorate inflammation-associated diseases. Below, their potential applications are highlighted.

### 6.1. Modulating the Immune System to Treat Immunological Disorders

*Trichinella spiralis* ES products are characterized by strong immunoregulatory properties that enable modulation of the host’s immune system and thus ensure its long-term survival [31]. It is characterized by a Th2 phenotype with induction of regulatory T cells (Tregs), alternatively activated macrophages (AAMs), regulatory dendritic cells (DCregs), and increased expression of immunoregulatory factors (IL-10 and TGF-β) [31,64,65,66]. The immunomodulatory effects of *T. spiralis* and their implications for allergic and autoimmune diseases have been the subject of extensive and long-standing research [65,67,68,69,70,71,72,73].

Recently, these regulatory properties have been largely attributed to *Ts*-EVs in ES products. Studies on the properties of *Ts*-EVs highlighted their role in most of the previously observed immunomodulatory effects of *T. spiralis* infection or their ES products. Kosanović et al. [30] were the first to point to the potential immunomodulatory role of *Ts*-ML-EVs. They investigated their regulatory response using a peripheral blood mononuclear cell (PBMC) stimulation assay and observed an increase in IL-10 and IL-6 levels and a decrease in the production of IL-17a levels in culture supernatants of PBMC incubated with different concentrations of *Ts*-ML-EVs in a manner similar to that of the whole ES L1 products from which they were isolated.

Glamoclija et al. [35] showed that *Ts*-EVs possess the potential to promote the generation of tolerogenic DCs and subsequently polarize T cell responses towards Th2 and Treg responses. The most prominent effect of *Ts*-EV-treated DCs is the induced expansion of conventional CD4^+^ CD25^+^ Foxp3^+^ Treg cells and unconventional type 1 regulatory cells (Tr1) with a high production of regulatory cytokines IL-10 and TGF-β and low production of Th1 cytokines, namely IL-12, IFN-γ, IL-17, and IL-23. Moreover, *Ts*-EVs-treated DCs showed a high expression of genes associated with the tolerogenic status of DCs such as *mTOR*, *AhR*, and *NFkB2*; *RelB*, *SOCS1*, and *SOCS3*; and *IDO*.

Interestingly, these tolerogenic properties of *Ts*-EVs-treated DCs were retained even after challenge with a pro-inflammatory stimulus (lipopolysaccharides from *Escherichia coli* and IFN-γ). Therefore, *Ts*-EVs induce immune regulatory mechanisms that modulate the host’s immune response not only to itself, but also to other foreign or self-antigens which can result in the alleviation of inflammatory diseases [35]. Thus, EVs set the ground for developing safe helminth product-based therapeutics. Experimental studies have revealed some new potential applications of *Ts*-EVs for the treatment of some immunological disorders such as autoimmune diseases and allergies.

#### 6.1.1. Inflammatory Bowel Diseases (IBD)

*Ts*-EV immunoregulatory properties were investigated in trinitrobenzene sulfonic acid (TNBS)-induced colitis in mice (a model for Crohn’s disease). *Ts*-EVs were injected intraperitoneally three times (50 μg/mice) every 3 days before induction of colitis. *Ts*-EVs markedly reduced Th1 and Th17 cells and pro-inflammatory cytokine secretion (IL-1β, IFN-γ, and TNF-α), upregulated the expression of Th2 cytokines (IL-4 and IL-13), and induced Treg differentiation with an increase in the regulatory cytokines (IL-10 and TGF-β) in colon tissues and in mesenteric lymph nodes. Subsequently, intestinal inflammation and colitis were ameliorated through regulation of Th1/Th2 balance. Moreover, *Ts*-EVs enhanced intestinal barrier integrity through induction of the expression levels of occludin and zo-1 tight junction protein in colonic tissues [36].

In further studies on the effect of *Ts*-EVs, Gao et al. [74] investigated the prophylactic outcome of the intraperitoneal injection of *Ts*-EVs on dextran sulfate sodium (DSS) colitis (a model for ulcerative colitis). They observed an improvement in disease activity index and macroscopic and histopathological scores of the *Ts*-EVs group compared to those of the DSS group. *Ts*-EVs upregulated the expression of IL-4, IL-10, TGF-β, and IL-13. In addition, *Ts*-EVs inhibited the classically activated macrophage polarization and increased the infiltration of AAMs into the colon. In addition, *Ts*-EVs promoted intestinal tissue repair and improved the intestinal barrier integrity by competition for L-arginine (Arg), upregulation of Arg-1, and proline production. Similarly, Wu et al. [37] showed that *Ts*-EVs can inhibit the levels of IL-1β, iNOS, TNF-α, IL-6, and IL-23 in macrophages, and at the same time, they promote the levels of anti-inflammatory factors Arg-1 and TGF-β in macrophages, as well as inhibiting fibroblast activation. Altogether, these findings suggest that *Ts*-EVs display a potent immunomodulatory capacity that may be a potential strategy to manage IBD.

#### 6.1.2. Airway Hyperreactivity Diseases

Glamoclija et al. [75] explored the therapeutic potential of *Ts*-ML-EVs using an ovalbumin (OVA)-induced model of respiratory allergy. They demonstrated that intranasal delivery of *Ts*-EVs significantly alleviated airway inflammation and decreased lung infiltration with eosinophils. Moreover, *Ts*-EV suppressed the production of OVA-specific IgE- and Th2-mediated responses in sensitized mice. Notably, the EVs induced a systemic immunoregulatory response by expanding Treg cell populations and increasing IL-10 and TGF-β levels in both the lungs and spleen, while a localized increase in Tr1 cells within the lungs contributed to local immune modulation by inhibiting naïve and memory T cell responses. Therefore, these findings highlight *Ts*-EVs as a promising candidate for treating allergic inflammation.

### 6.2. Trichinella EVs as Potential Diagnostic and Prognostic Biomarkers

Parasite-derived EVs are structurally stable, rich sources of different molecules across diverse biofluids, including blood, saliva, urine, and cerebrospinal fluid (CSF) [76]. Due to their highly immunogenic cargo, they can serve as valuable biomarkers for the diagnosis of infectious diseases [77]. Furthermore, EVs provide a molecular snapshot of the parasite life cycle within the host. Notably, de Pontes et al. [78] established a positive correlation between EV concentration and infection intensity. Consequently, EV-based biomarkers represent a non-invasive approach for disease detection, prognosis, and therapeutic monitoring [12,79]. Given the increasing need for advanced diagnostics, EVs produced under Good Manufacturing Practice (GMP) protocols offer a promising frontier for clinical application [80].

The clinical diagnosis of human trichinellosis is challenging due to its non-specific clinical presentations. While muscle biopsy offers a definitive diagnosis, its utility is limited by its invasive nature and a high risk of false negatives during early-stage infections [81]. Although the International Commission on Trichinellosis (ICT) recommends serological testing to detect anti-*Trichinella* IgG against ML/ES antigens [28,82], these assays possess significant limitations including cross-reactivity with other nematodes, a delayed 3–4-week window before seroconversion, and low sensitivity in the early phases of the disease [83,84]. Furthermore, because antibody detection cannot reliably distinguish between active and past infections, the detection of circulating *Trichinella* antigens has emerged as a superior method for identifying current infections [85].

During the early stages of infection, *Ts*-ML-EVs and *Ts*-AW-EVs/miRNAs are released into the host’s bloodstream. Using quantitative PCR, Khueangchiangkhwang et al. [39] detected various miRNAs in *T. spiralis*-infected mice as early as one week post infection. Therefore, miRNA within *Trichinella*-secreted EVs can be a potential early-detection diagnostic tool for trichinellosis. Moreover, the host muscle cells themselves produce EVs that interact with satellite cells to stimulate their proliferation. The proteomic shifts in these muscle-derived EVs during infection may make them potential biomarkers for diagnosis. For instance, vimentin expression—an intermediate filament protein induced by *T. spiralis* NBL in muscle cells—was found to be significantly enriched in these EVs. Thus, it is suggested to be a novel candidate for the diagnosis of trichinellosis [86].

Interestingly, Ma et al. [87] characterized the profile of host circulating miRNAs in serum at 12, 18, and 30 days post infection (dpi) with *T. spiralis*. Significant miRNA alterations were observed during the early pre-encapsulation stages (12–18 dpi) when serological tests were still negative. They further identified distinct expression patterns of mmu-miR-467a-3p and mmu-miR-467d-3p that reached a peak at 30 dpi, whereas mmu-miR-376b-3p and mmu-miR-664-3p increased significantly at 18 dpi and then declined. In addition, mmu-miR-292a-5p expression gradually decreased from 12 to 30 dpi. Because these circulating miRNAs target specific signaling pathways involved in host–parasite interactions and vary in different phases of *T. spiralis* infection, they represent highly sensitive biomarkers for trichinellosis, especially in the early phase of infection.

Recently, Li et al. [43] used LC-MS/MS technology combined with bioinformatic analysis to characterize the proteomic profile of *Ts*-ML-EVs. They identified a candidate molecule, *Ts*-TTPA, a member of the S1 peptidase family containing a characteristic trypsin domain. Upon evaluation of the recombinant *Ts*-TTPA protein as a potential diagnostic antigen, it was found to detect anti-*Trichinella* IgG levels in swine serum as early as 7 dpi with no cross-reactivity with other common swine parasites. These findings suggest that EV-derived proteins, such as *Ts*-TTPA, are highly effective antigens for the early detection of trichinellosis.

Although not well documented, many of the previously mentioned proteins and miRNA cargo in *Ts*-EVs might be potential diagnostic markers. For instance, among the expressed miRNAs in *Ts*-NBL-EVs, let-7-5p exhibits the highest expression levels during early stages of *Trichinella* infection [40] and it is similarly expressed in *Ts*-ML-EVs [37]. Celik et al. [88] reported that let-7-5p serves as a biomarker for the early diagnosis of *Echinococcus granulosus* infection in dogs. Therefore, *Ts*-EVs offer significant potential as circulating biomarkers due to their abundance, structural stability, and ease of accessibility. Moreover, they represent an active biological process and maintain a proteomic profile that closely reflects their cell of origin.

### 6.3. Trichinella EVs as Potential Candidates for Vaccine Development

Single-antigen vaccines are often inadequate against the complex, multi-stage life cycle of *T. spiralis*. Therefore, multiepitope vaccines against *T. spiralis* were suggested as a better strategy [89]. EVs are potential vaccine candidates because of their protective lipid bilayer that allows slow release of antigens and protects them from degradation [90]. Moreover, structural proteins such as annexins and syntenin-1 identified in the EVs of many helminths could represent good candidates for vaccines because these homogenous proteins are not expressed in humans [91]. Importantly, the nano size of EVs ensures that they are efficiently internalized by antigen-presenting cells, thereby significantly boosting their antigenicity [92].

To date, no clinical trials have yet been reported; however, few experimental studies have investigated *T. spiralis*-derived EVs as novel vaccine candidates with a great potential for biotechnological development in the near future. Ashour et al. [32] evaluated *Ts*-ML-EVs as a potential vaccine candidate. They immunized experimental mice by subcutaneous injection of *Ts*-ML-EVs in two doses, one week apart, and then the mice were challenged with *T. spiralis* infection one week later. They demonstrated that *Ts*-ML-EVs induced a protective, mixed Th1/Th2 immune response with obvious Th1 polarization as well as increased serum *T. spiralis*-specific IgG resulting in a marked reduction in the numbers of adult worms (78.5%) and muscle larvae (~97%). Similarly, Gao et al. [33] used subcutaneous injection of *Ts*-ML-EVs in three doses, two weeks apart. Two weeks after the last immunization, experimental animals were challenged with *T. spiralis* infection. The intestinal adult worm burden of the *Ts*-ML-EV group was reduced by 23.4% and the ML burden was reduced by 43.7% in the immunized groups, suggesting that *Ts*-ML-EVs contain antigens that can effectively reduce the transmission and pathology of *T. spiralis*. They reported increased serum levels of IgM, IgG, and IgE in addition to an induced mixed response of Th1 and Th2.

Collectively, immunization with *Ts*-ML-EVs induces immune responses that confer resistance to *T*. *spiralis* through different proposed mechanisms: the rapid expulsion of adult worms from the intestine or delaying the larval invasion of the enteric mucosa, and the destruction of the AW and NBL larvae by antibody dependent cellular cytotoxicity (ADCC) or via the cytotoxic killing effect of eosinophils and granulocytes in immunized mice [32,33]. A notable finding in these vaccination models is the ability of EVs to regulate immune responses, thereby establishing a balanced Th1/Th2 profile and ameliorating the tissue pathology in the intestine and muscles. Whether *Ts*-AW-EVs could induce a protective immune response against *T*. *spiralis* awaits further investigation.

## 7. Limitations and Pitfalls

Despite the great potential of *Ts*-EVs, several significant challenges must be addressed before clinical implementation. Current methodologies lack the reproducibility and cost-effectiveness required for large-scale production [93,94,95,96]. Specifically, there are no standardized isolation and purification protocols or efficient drug-loading systems tailored for clinical-scale applications. These challenges represent critical barriers as Amruta et al. [97] suggested that any EV damage during isolation, drug loading, and/or storage may cause systemically administered EVs to exit the circulation within minutes and accumulate in the liver and spleen. Furthermore, the commonly used detection methods such as ELISA and Western blotting lack the necessary sensitivity for low-volume samples [98].

Moreover, much of the research has relied on in vitro parasite cultures rather than non-invasive biofluids such as plasma [99]. The yield of EVs in culture medium is usually low and insufficient for clinical translation [100]. As mentioned above, almost all studies focused on ML as a source of EVs. Very few used AW or NBL as a source of *Ts*-EVs. Different cargo detected in EVs isolated from different stages of *T. spiralis* can show different functions. Thus, further profiling and functional studies—particularly those aimed at identifying the core set of bioactive molecules—are essential to validate their therapeutic and diagnostic utility.

In almost all studies regarding *Ts*-EVs, information on the composition of *Ts*-EVs is limited. Even in the studies that performed proteomic analysis of *Ts*-EVs [33,39], they reported that the association of those proteins with *Ts*-EVs requires further confirmation. Furthermore, the existing studies on *Ts*-EVs cargo do not distinguish whether the contained proteins originate from *T. spiralis* or from the host.

Widespread clinical application of EVs remains constrained owing to multiple safety issues. EV preparations are considered to be a type of biopharmaceutical preparation from cells that may include co-isolated impurities and may be contaminated with culture-medium-derived components/reagents such as serum proteins, microorganisms, endotoxins, and virus particles or their fragments. Because EVs have high target specificity, it is unlikely to cause off-target toxicity. However, it may occur from these impurities. Thus, it is important to establish strict quality-controlled manufacturing processes to minimize the inclusion of components other than the intended EVs. Risks due to undesirable immune responses such as allergy or rejection are assumed with EVs derived from non-human sources, which is the case in parasite-derived EVs. Although there is no definitive evidence, the risk of carcinogenicity due to contamination with cell-derived DNA needs to be considered [101,102,103].

Although delivering miRNA via EVs is a promising solution that can overcome its instability and susceptibility to degradation in circulation, several challenges continue to delay their clinical development such as immunological off-target effects caused by partial complementarity with non-targeted transcripts’ “too many targets for miRNA effect” (TMTME), which may lead to unintended gene regulation of critical cellular pathways. Moreover, delivering miRNAs at excessively high concentrations that overwhelm the cellular machinery leading to miRNA-dependent cellular toxicity is another critical safety concern in miRNA -based therapeutics [104,105,106].

## 8. Conclusions and Future Perspectives

With the discovery of EVs and their importance in cellular communication, many studies have focused on parasite-derived EVs. They contain various parasitic factors and signaling molecules to modify the hostile microenvironment of their hosts for their benefit [107]. Over the past few years, *Ts*-EVs have attracted a significant interest that provides much information about their role in pathogenicity and immunomodulation of the host immune response with their potential applications. We collected almost all of the studies on *Ts*-EVs to determine their current status. Despite these insights, in-depth research into the whole picture and all the functions of EVs in *T. spiralis* infection is yet to be conducted, and much research is still needed in order to harness EVs for use in human clinical medicine.

Accurate and reliable methods for EV purification and characterization are crucial for using EVs as a tool for diagnosis and therapy. Exploring the composition of specific cargo molecules carried by *Ts*-EVs is critical not only to clarify their roles during *T. spiralis* infection and host–parasite communication but also to be used as a parasite-specific biomarker and as a rich molecular source for vaccine development [108]. Such knowledge would further enable the design of bioengineered synthetic vesicles or formulations that mimic the beneficial properties of *Ts*-EVs, and may lead to the development of scalable, high-quality, and safe biologics for clinical applications [75].

A great effort has been made by the International Society for Extracellular Vesicles (ISEV) that continuously updates the Minimal Information for Studies of Extracellular Vesicles (MISEV) guidelines for the standardization of procedures for EV isolation and characterization [109]. It is now the role of the researchers to stick to them to obtain reliable findings. Multiple new technologies have been developed in the past decade for EVs isolation and characterization. Micro/nanofluidic technology with affinity capture offers precise EV enrichment results in EVs that are highly purified compared to conventional methods [110]. Moreover, detection of a single EV is now possible by nanoplasmonic sensing technology for EV detection that allows sensitive multiplexed quantifiable detection of EVs through a simple assay, thus improving the potentiality and accuracy of EVs as diagnostic markers and for the prediction of treatment outcomes [12].

EVs have been tested as a drug delivery vehicle for the delivery of different molecules including drugs, which provide potential advantages over traditional synthetic delivery vehicles, such as liposomes and nanoparticles (reviewed in Meng et al. [111]). The nano size of EVs makes it one of the ideal carriers for the delivery of active molecules and drugs. They have successfully been applied in cancer therapy, inflammatory modulation, and immune response regulation [112,113,114]. Although their effectiveness has not yet been fully investigated, parasite-derived EVs may represent a promising carrier for drug delivery as they provide several advantages, such as easy bypass of cellular barriers [115] and delivered drug protection [116].

Regarding the treatment options of trichinellosis, benzimidazole derivatives such as albendazole, the most commonly used drug, have a poor bioavailability and hence it affects only the AW in the intestine with limited efficiency in eliminating ML [117]. Thus, based on EV tissue tropism to their cells of origin [118], we hypothesize that if albendazole, for example, is loaded on *Ts*-EVs, it may gain better access and reach ML more effectively. Yet, extensive studies on the pharmacokinetics of drug-loaded EVs, determining the optimal dosage for maximizing benefits while minimizing side effects and the availability of efficient drug loading systems for clinical-scale production, are required [95,119,120]. The stability and durability of EVs ensure the execution of such a mission with ease, and via genetic engineering techniques, we should be able to produce large quantities of EVs, all displaying the same molecular and physiological signature and all containing a single agent, or more likely, a cocktail of therapeutic molecules.

In summary, *Ts*-EVs hold promise as candidates for diverse future applications, including novel diagnostic biomarkers, effective vaccines, as well as therapeutic uses. Of equal importance is the assumption that these EVs carry molecules that facilitate their transfer into specific cells, for instance, intestinal and muscle cells in the case of *Trichinella*. Identification of these molecules may present a modality of targeted therapy where specific cells are selected and other normal cells are spared.

## Figures and Tables

**Table 1:** An overview of stage-specific *Trichinella* EVs and their characteristics.

Source	Isolation Method	Size (Diameter)	Characterization	References
Muscle larvae (*Ts*-ML-EVs)	Differential centrifugation	30–80 nm (TEM)	Two predominant protein bands at 49 and 53 kDa with less prominent bands at 32, 44, 65, and >200 kDa (SDS-PAGE); β-actin and 7C2C5 protein expression (Western blot).	[30]
	Ultracentrifugation	30–150 nm (TEM), 50–250 nm, peak at 133 nm (NTA)	CD63 and enolase expression (Western blot).	[36]
	Ultracentrifugation	24–86 nm (TEM)	Prominent protein band at 49 kDa (SDS-PAGE); CD63 and CD73 expression (beads-based flow cytometry).	[32]
	Ultracentrifugation	˂200 nm (TEM)	EV proteins mainly within 45–70kDa kDa (SDS-PAGE); enolase expression (Western blot) and 753 total proteins defined (proteomic analysis).	[33]
	Ultracentrifugation	60–120 nm (TEM & NTA)	CD63 expression (Western blot).	[34]
	Ultracentrifugation	30–300 nm (peak at 138 nm) (TEM & NTA)	EVs contained 64 miRNAs (miRNA-1-3p and let-7-5p were the most highly expressed) (miRNA sequencing).	[37]
	Differential centrifugation and ultrafiltration	30–80 nm (TEM), average size 156 ± 74 nm (NTA)	Induced regulatory immune responses in vitro.	[35]
Adult worms (*Ts*-AW-EVs) and muscle larvae (*Ts*-ML-EVs)	Ultracentrifugation	Up to 150 nm (TEM & NTA)	Unencapsulated small RNAs, peaking at 23 nt in length (high-throughput sequencing).	[38]
	Ultracentrifugation	30–50 nm (electron microscopy and flow cytometry)	AW-EVs and ML-EVs share 168 common proteins; ML-EVs had 62 stage-specific proteins and AW-EVs had 92 stage-specific proteins (LC-MS/MS).	[39]
Newborn larvae (*Ts*-NBL-EVs)	Differential ultracentrifugation	~101 nm (TEM & NTA)	Prominent protein band at ~65 kDa (Western blot), expression of 231 proteins such as 14-3-3 protein, and ENO1 protein. CD63 and CD81 not detected (MS-based proteomics). Identification of 183 miRNAs in EVs (miRNA sequencing).	[40]

NTA, nanoparticle tracking analysis; SDS-PAGE, sodium dodecyl-sulfate polyacrylamide gel electrophoresis; TEM, transmission electron microscope.

**Table 2:** The most frequently identified protein cargo in *Trichinella spiralis* EVs and their potential functions.

Protein Cargo	Potential Functions	References
CD63 (a tetraspanin protein)	Surface markers of EVs but their specific function has not yet been defined.	[32,34,43]
CD81 (a tetraspanin protein)		[43]
Heat shock protein 70 (HSP70)	Crucial for parasite survival under different stressors including oxidative stress of the host immune response [44].	[30]
Enolase (metabolic enzyme)	Multifunctional protein essential for parasite survival and invasion of the host tissues via degradation of the extracellular matrix [45].	[33,36,40,43]
Serine proteases (e.g., *Ts*-TTPA protein)	Involved in the process of *T. spiralis* invasion of intestinal epithelium of the host.	[34]
14-3-3 protein	A conserved regulatory molecule that plays key roles in several eukaryotic biochemical processes, such as signal transduction, transport, regulation, cell differentiation, and cell survival [46].	[40,43]
7C2C5 protein (glycoprotein triad containing 45, 49, and 53 kDa)	Induction and polarization of immunomodulatory responses [47].	[30]
Actin-5C	Cytoskeletal protein.	[39]
β-actin	Involved in nurse cell formation [48].	[30]
Myogenin (*MyoG*)	Nurse cell formation.	[39]
Vascular endothelial cell growth factor (VEGF)	Angiogenic molecule increased during nurse cell formation and encapsulation in the muscle.	[39]

## Data Availability

The original contributions presented in this review are included in the article.

## Abbreviations

AAMs, alternatively activated macrophage; ADCC, antibody-dependent cellular cytotoxicity; AW, adult worm; Bregs, regulatory B cells; ES, excretory–secretory; intercellular adhesion molecule 1 (ICAM1); ML, muscle larva; MS, mass spectrometry; miRNAs, microRNAs; NBL, newborn larva; PBMC, peripheral blood mononuclear cell; TGF-β, transforming growth factor–β; Tregs, regulatory T cells; *Ts*-AW-EVs, *T. spiralis* adult worm EVs; *Ts*-EVs, *Trichinella spiralis* extracellular vesicles, *Ts*-ML-EVs, *T. spiralis* muscle larva EVs; *Ts*-NBL-EVs, *T. spiralis* newborn larva EVs; VEGF, vascular endothelial cell growth factor.
